# A systematic review of minor phytocannabinoids with promising neuroprotective potential

**DOI:** 10.1111/bph.15185

**Published:** 2020-09-01

**Authors:** Nicole L. Stone, Alexandra J. Murphy, Timothy J. England, Saoirse E. O'Sullivan

**Affiliations:** ^1^ Division of Medical Sciences and Graduate Entry Medicine, School of Medicine University of Nottingham, Royal Derby Hospital Derby UK

**Keywords:** Alzheimer's, epilepsy, Huntington's, neurodegeneration, neuroprotection, phytocannabinoids

## Abstract

Embase and PubMed were systematically searched for articles addressing the neuroprotective properties of phytocannabinoids, apart from cannabidiol and Δ^9^‐tetrahydrocannabinol, including Δ^9^‐tetrahydrocannabinolic acid, Δ^9^‐tetrahydrocannabivarin, cannabidiolic acid, cannabidivarin, cannabichromene, cannabichromenic acid, cannabichromevarin, cannabigerol, cannabigerolic acid, cannabigerivarin, cannabigerovarinic acid, cannabichromevarinic acid, cannabidivarinic acid, and cannabinol. Out of 2,341 studies, 31 articles met inclusion criteria. Cannabigerol (range 5 to 20 mg·kg^−1^) and cannabidivarin (range 0.2 to 400 mg·kg^−1^) displayed efficacy in models of Huntington's disease and epilepsy. Cannabichromene (10–75 mg·kg^−1^), Δ^9^‐tetrahydrocannabinolic acid (20 mg·kg^−1^), and tetrahydrocannabivarin (range 0.025–2.5 mg·kg^−1^) showed promise in models of seizure and hypomobility, Huntington's and Parkinson's disease. Limited mechanistic data showed cannabigerol, its derivatives VCE.003 and VCE.003.2, and Δ^9^‐tetrahydrocannabinolic acid mediated some of their effects through PPAR‐γ, but no other receptors were probed. Further studies with these phytocannabinoids, and their combinations, are warranted across a range of neurodegenerative disorders.

**Abbreviations**
3‐NP: 3‐nitropropionic acid
6‐OHDA: 6‐hydroxydopamine
Δ^9^‐THCA: Δ^9^‐tetrahydrocannabinolic acid
Δ^9^‐THCV: Δ^9^‐tetrahydrocannabivarin
BBB: blood–brain barrier
CBC: cannabichromene
CBCA: cannabichromenic acid
CBCV: cannabichromevarin
CBDA: cannabidiolic acid
CBDV: cannabidivarin
CBDVA: cannabidivarinic acid
CBG: cannabigerol
CBGA: cannabigerolic acid
CBGV: cannabigerivarin
CBGVA: cannabigerovarinic acid
CBM: cannabis‐based medicine
CBN: cannabinol
CNCVA: cannabichromevarinic acid
MS: multiple sclerosis
RTT: Rett syndrome
TBZ: tetrabenazine
VMAT: vesicular monoamine transporter

## 1 INTRODUCTION

According to the World Health Organization (WHO), neurodegenerative diseases will be the second most prevalent cause of death by 2040 (Gammon, 2014). The cellular mechanisms of these diseases typically overlap with neuronal dysfunction and a common thread is neuronal cell death, regardless of definitive clinical presentations. Typically, neurodegenerative diseases are categorized as amyloidoses, which includes Alzheimer's disease and British familial dementia; synucleinopathies, which includes Lewy body disorders such as Parkinson's; and proteinopathies, which includes amyotrophic lateral sclerosis and tauopathies (Kovac, 2018). Other common neurological disorders include epilepsy and stroke, characterized by recurring, unprovoked seizures and vascular pathology, respectively. Recently, stroke was reclassified as a neurological disease by the International Classification of Disease (ICD) 11, highlighting that while strokes predominantly have a vascular origin, the neurological consequences are often severe (Shakir, 2018).

Current treatments for neurodegenerative and neurological conditions are often limited and usually rely on managing symptoms rather than having a significant effect on delaying disease progression (Kiaei, 2013). For example, Huntington disease is managed with tetrabenazine (TBZ) 75–200 mg per day to alleviate chorea (involuntary movement), but because it acts as a vesicular monoamine transporter (VMAT) inhibitor, interfering with both 5‐HT and dopamine degradation, patients can develop neuropsychiatric symptoms along with other side effects (Hayden, Leavitt, Yasothan, & Kirkpatrick, 2009; Kaur, Kumar, Jamwal, Deshmukh, & Gauttam, 2016; Wyant, Ridder, & Dayalu, 2017). Other first‐line treatments, for example, l‐Dopa in Parkinson's disease, often cause side effects and do not delay disease progression. Finally, cholinesterase inhibitors such as donepezil are only minimally effective in improving cognition for the treatment of Alzheimer's disease. In light of this, there is clearly an urgent need to develop new therapies with more tolerable side effect profiles to combat these debilitating conditions and increase the quality of life of the ageing population.

Over 120 different phytocannabinoids have been isolated from *Cannabis sativa* (ElSohly & Gul, 2015). Of these, Δ^9^‐tetrahydrocannabinol (Δ^9^‐THC) and cannabidiol (CBD) are the most abundant and widely studied. Δ^9^‐THC is responsible for the psychoactive effects of cannabis, which are mediated through the cannabinoid CB_1_ receptor (Pertwee, 2008). Δ^9^‐THC also interacts with other targets including transient receptor potential (TRP) channels, the orphan G‐protein receptor, GPR55, and peroxisome proliferator‐activated receptors (PPARs; Pertwee & Cascio, 2015). CBD has also been shown to modulate a wide range of pharmacological targets including 5‐HT_1A_ receptors, PPARγ and TRPV1 channels, but has no psychotropic effects because it does not activate central CB_1_ receptors (see Ibeas Bih et al., 2015, and Russo & Marcu, 2017). Interaction with these targets has given CBD status as a neuroprotectant, anti‐inflammatory agent and antioxidant (Fernandez‐Ruiz et al., 2013; Maroon & Bost, 2018). These features, along with its favourable safety profile in humans (Millar et al., 2019; World Health Organization, 2017) has made CBD, in many respects, a more desirable drug candidate than Δ^9^‐THC. CBD has shown promise in several animal models of neurodegeneration as well as clinical trials for Parkinson's, Alzheimer's and amyotrophic lateral sclerosis (Iuvone, Esposito, de Filippis, Scuderi, & Steardo, 2009). Furthermore, a fixed combination of CBD and Δ^9^‐THC (1:1) is currently licenced by GW Pharmaceuticals under the brand name Sativex® to treat pain and spasticity associated with multiple sclerosis (MS), and Epidiolex® (pure CBD) is licensed to treat Lennox–Gastaut syndrome and Dravet syndrome, which are severe forms of childhood epilepsy. Other cannabis‐based medicines (CBMs) are also under development. GW Pharmaceuticals has four compounds (structures are not disclosed) in the pipeline for neurological conditions including glioblastoma, schizophrenia and neonatal hypoxic‐ischaemic encephalopathy (GW Pharmaceuticals, 2019).

Phytocannabinoids are highly unique compounds, they are promiscuous in action, modulating a range of pharmacological targets as well as exhibiting high antioxidant capability due to their phenolic structures and the presence of hydroxyl groups (Borges et al., 2013; Hampson, Grimaldi, Axelrod, & Wink, 1998; Yamaori, Ebisawa, Okushima, Yamamoto, & Watanabe, 2011). These features, along with their lipophilicity and ability to act as anti‐inflammatory agents, makes them desirable therapeutic candidates for the treatment of CNS disorders, as they can effectively cross the blood–brain barrier (BBB), modulate the immune response, and target the many aspects of neurodegeneration (Deiana et al., 2012). These characteristics have been well established for Δ^9^‐THC and CBD but are less well known for some of the minor constituents of the plant. Thus, in order to understand the full therapeutic potential of *Cannabis sativa*, the pharmacology of the lesser‐known components of the plant should be elucidated (Turner, Williams, Iversen, & Whalley, 2017). Given the wide‐ranging neuroprotective effects of Δ^9^‐THC and CBD already established, it is not unreasonable to suggest other phytocannabinoids may exhibit similar or more potent neuroprotective properties. Therefore, the aim of this systematic review was to collate all available data on the neuroprotective effects of Δ^9^‐tetrahydrocannabinolic acid (Δ^9^‐THCA), Δ^9^‐tetrahydrocannabivarin (Δ^9^‐THCV), cannabidiolic acid (CBDA), cannabidivarin (CBDV), cannabichromene (CBC), cannabichromenic acid (CBCA), cannabichromevarin (CBCV), cannabigerol (CBG), cannabigerolic acid (CBGA), cannabigerivarin (CBGV), cannabigerovarinic acid (CBGVA), cannabichromevarinic acid (CBCVA), cannabidivarinic acid (CBDVA), and cannabinol (CBN). These phytocannabinoids were selected based on their abundance in the plant, ease of synthesis, efficacy in other fields (e.g., as anticancer agents or treatments for inflammatory bowel disease), and similarities in their structure to CBD and Δ^9^‐THC (which have already shown promise as a neuroprotectants and displayed safety in humans) and are therefore more likely to have neuroprotective potential and exhibit human translatability.

## 2 METHODS

### 2.1 Data sources and search strategy

An electronic search was conducted using the search engines PubMed and Embase from its inception to June 2019. This was carried out in accordance with the PRISMA (Preferred Reporting Items for Systematic Reviews and Meta‐Analyses) guidelines (Moher, Liberati, & Tetzlaff, 2009; Shamseer et al., 2015; Tóth, Schumacher, Castro, & Perkins, 2010) Search terms included Δ^9^‐tetrahydrocannabinolic acid, Δ^9^‐tetrahydrocannabivarin, cannabidiolic acid, cannabidivarin, cannabichromene, cannabichromenic acid, cannabichromevarin, cannabigerol, cannabigerolic acid, cannabigerivarin, cannabigerovarinic acid, cannabichromevarinic acid, cannabidivarinic acid and cannabinol (and their corresponding abbreviations), phytocannabinoids, neurovascular unit, pericytes, neurons, astrocytes, human brain microvascular endothelial cells, brain, neuroinflammation, hyperexcitability, neurodegeneration, Huntington's, Alzheimer's, Parkinson's, epilepsy, and stroke. Two independent reviewers carried out the searches by November 2019, and the reference lists of the final papers were hand searched for any additional studies.

### 2.2 Eligibility and exclusion criteria

Conference abstracts and review articles were excluded. No restrictions were applied to type of study, publication year, or language. Inclusion criteria were as follows: an original, peer reviewed article that involved the application of emerging phytocannabinoids in an in vivo or in vitro model of neurodegeneration or neuronal damage. Studies that looked at two derivatives of CBG, known as VCE‐003 or VCE‐002.3 were also included because current research is focused on these compounds, based on their increased affinity for PPARγ. Studies that assessed CBD, Δ^9^‐THC, Δ^9^‐THC:CBD 1:1 (Sativex®), or similar combinations of phytocannabinoids (i.e., different ratios of phytocannabinoids) were excluded from this review. After duplicates and irrelevant articles were removed, the full text was obtained for the remaining articles, and studies were examined for data regarding Δ^9^‐THCA, Δ^9^‐THCV, CBDA, CBDV, CBC, CBCA, CBCV, CBG, CBGA, CBGV, CBGVA, CBCVA, CBDVA, and CBN application in an in vitro and/or in vivo model of neuroprotection or neuronal damage. Dose and route of administration were extracted from in vivo studies and where possible range and average were calculated. If studies reported mechanistic data, this was also described in Section 3.

## 3 RESULTS

**FIGURE 1:**
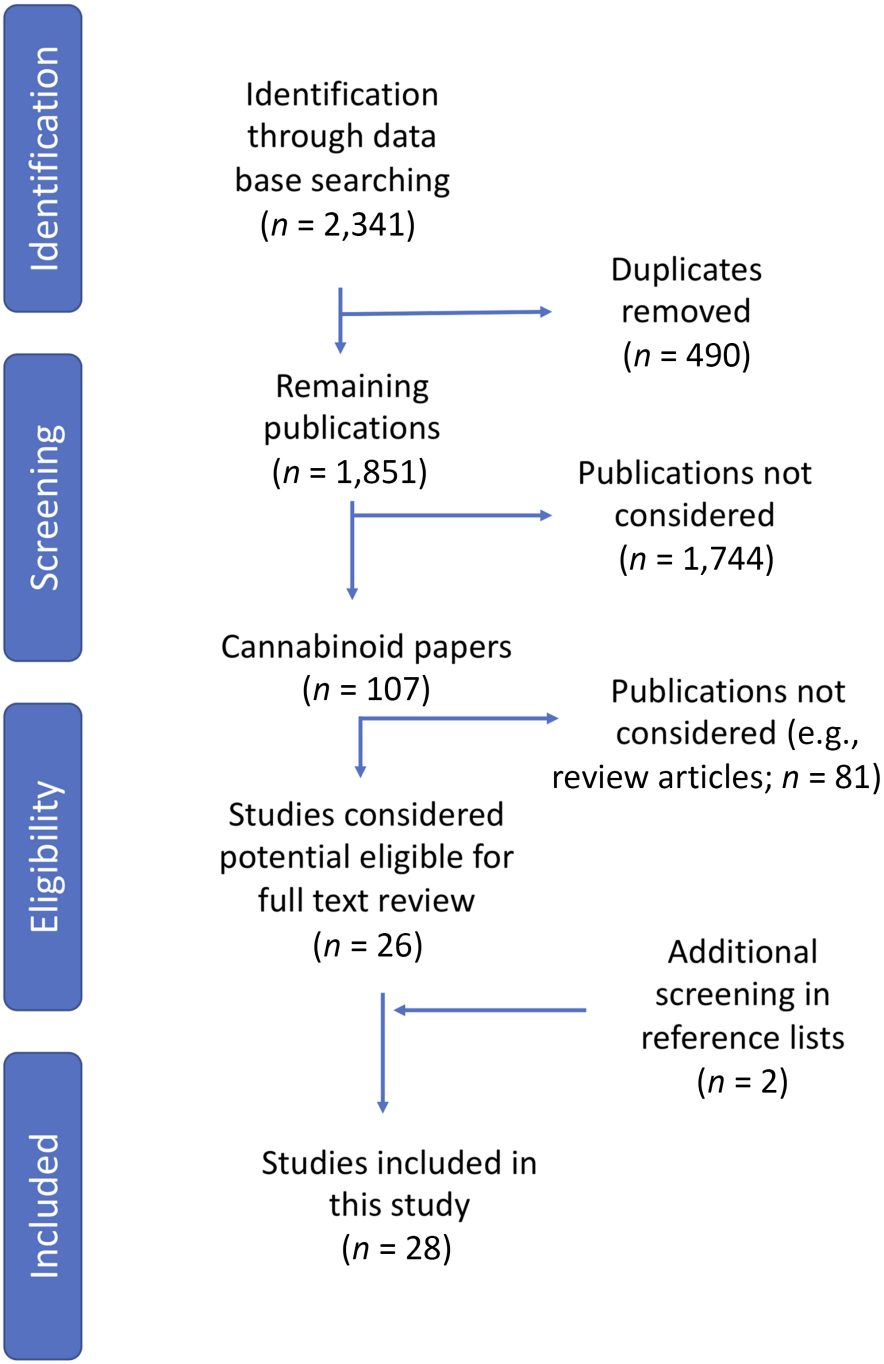
Overview of methodology used in the search process, identification, screening, eligibility, and inclusion

The preliminary search retrieved 2,341 studies, which after duplicates were removed left 1,851. A total of 107 cannabinoid studies were retrieved; once exclusion criteria were applied, 26 articles were considered to be potentially relevant and their full texts obtained. After additional screening (including reviewing reference lists for any potential studies), 28 studies were included in this review; see Figure 1. Table 1 summarizes the *in vitro* data included in this review, and Table 2 summarizes the *in vivo* data.

**TABLE 1:** Summary of included *in vitro* studies

Phytocannabinoid	Compound	Concentration/Incubation period	Neuro model	Cells used	*n* number	Results	Study
Cannabigerol (CBG)	Cannabigerol derivative VCE‐003.2	500 nM for 21 days	Huntington's disease	Mouse embryonic stem cells (R1 line)/P19 neurospheres	*n* = 3	VCE‐003.2 increased CTIP‐2 positive cells, promoted neuronal like‐differentiation and significantly larger P19 neurospheres versus vehicle treated cells (*P* < 0.01)	Aguareles et al. (2019)
	Cannabigerol derivative VCE‐003	1, 5, 10 μM (human T‐cells). 1 and 2.5 μM (RAW 264.7 cells) for 3 days post stimulation	Autoimmune Encephalomyelitis to model multiple sclerosis (MS)	Jurkat, BV2 RAW 264.7 cells. Human peripheral T‐cells	*n* = 3^a^	1 μM reduced expression of iNOS in BV2 microglial cells. Antagonists AM630 (CB_2_) and GW9662 (PPARγ) blocked these effects. Prevented T cell division at 1 and 5 μM and inhibition of the release of all soluble mediators (T‐cells)	Carrillo‐Salinas et al. (2014)
	Cannabigerol derivatives: VCE‐003 and VCE‐003.2	1–50 μM (N2a) for 24 h 50 nM–50 μM (HiB5) 30, 10, and 3 μM for 6 h	Huntington's disease	(N2a cells/HiB5 cells) Immortalized striatal neuroblasts expressing huntingtin/mutant repeats	*n* = 3^a^	VCE‐003.2 improved cell viability (10 and 25 μM) and prevented excitotoxicity in N2a cells. VCE‐003.2. Reduced the number of cells with aggregates (neuroblasts) and improved neuronal viability post serum deprivation	Diaz‐Alonso et al. (2016)
	VCE‐003 cannabigerol quinone derivative	0.1‐, 1‐, 10‐, and 25‐μM CBG/VCE‐003 (HTT cells, 24 h) (microglia, 18 h; hippocampal cells; mice treated 15 days 5 mg·kg^−1^ i.p. VCE‐003^b^)	Multiple sclerosis	HEK293 cells and primary microglial cells. HT22 mouse hippocampal cells	*n* = 3^a^	VCE‐003 protected neuronal cells from excitotoxity. Reduction in IL‐1β, IL‐6, TNF‐α, PGE_2_, and MIP‐1‐α in microglia (1, 10, and 25 μM) VCE‐003 ameliorated MS symptoms induced by TMEV	Granja et al. (2012)
	VCE‐003.2 cannabigerol derivative	BV2 cells 5 μM VCE‐003.2 for 21 h. VCE‐003.2 (M‐213 cells) Vehicle (0.1% DMSO) versus 0.1, 0.5, and 1 μM for 40 h	Parkinson's disease model induced by LPS (conditioned medium from BV2 cells added to M‐213 cells)	Mouse microglial BV2 cells. M‐213 (striatal cell line) neuronal cells	BV2 cells: *n* = 14, 7 repeats	In BV2 cells, VCE‐003.2 significantly decreased TNF‐α COX‐2 and iNOS mRNA. Attenuated TNF‐α and IL‐1β secreted in medium of BV2 cells (5 μM)	Garcia et al. (2018)
	Cannabigerol	MTT assay: 1, 2.5, 5, 7.5, 10, 12.5, 15, and 20 μM pretreated 24 h. NSC‐34: pretreated with 7.5 μM	Neuroinflammation—medium from LPS stimulated macrophages	NSC‐34 motor neurons	*n* = 3 repeats	CBG at 2.5 and 7.5 μM increased cell viability approximately 20% compared to control. CBG pretreatment inhibited apoptosis and reduced; IL‐1β, TNF‐α, INF‐Y (NSC‐34 motor neurons). CBG restored decreased Nrf2 levels	Gugliandolo et al. (2018)
	Cannabidiol* and cannabigerol	Electrophysiology: 1/10 μM 20 min. hNAv cells: 1 nM–200 μM for 100 s	PTZ seizures	Transverse hippocampal slices, SH‐SY5Y, hNAv cell lines	SH‐SY5Y—*n* = 6 mouse cortical neurons *n* = 8 hNAv *n* = 3	10‐μM CBG significantly reduced peak Nav current in SH‐SY5Y cells and mouse cortical neurons. CBG was also effective as a low affinity Nav channel blocker.	Hill et al. (2014)
	Cannabigerol derivative VCE‐003.2	0.1, 0.5, 1, and 5 μM added 1 h prior to LPS, for 24 h	Amyotrophic lateral sclerosis	Astroglial cells (mutant SOD1 mice)	*n* = 4, 6 samples per group	VCE.003.2 at 1 and 5 μM attenuated levels of TNF‐α and IL‐1β, elevated due to LPS stimulation	Rodrígueuz‐Cueto et al. (2018)
	Cannabigerol	6 h—supplementary information cannot be accessed	Huntington's disease	Immortalized striatal progenitor cells: STHdh^Q7/Q7^ and STHdh ^Q111/Q111^ cells	*n* = 3 repeats	CBG dose‐dependently activated PPARγ	Valdeolivas et al. (2015)
	Cannabigerol	1‐μM 24‐h ATP assay/viability and differentiation for 2 days	Neuroprotection	Adult neural stem cells/progenitor cells (NSPC)	*n* = 6	CBG had no significant effect on any of the endpoints measured.	Shinjyo & Di Marzo (2013)
Cannabidivarin (CBDV)	Cannabidivarin	1, 10, and 100 μM 30 min after epileptiform activity for 30 min	Epilepsy‐spontaneous local field potentials (LFPs)	Transverse hippocampal slices male/female Kyoto rats	*n* > 5 slices from *n* > 5 animals	CBDV decreased amplitude and duration of LFPs and increased Mg2 + free induced LFPs frequency (>10 μM).	Hill et al. (2012)
	Cannabidivarin (+CBD)	3, 10, 30 μM 30–40 min after control readings for 1 min	Epilepsy	HEK cells (HEK293) transfected with TRPV1, TRPV2, and TRPA1.	*n* = 4	CBDV was anticonvulsant, and TRPV1 antagonist capsazepine blocked this effect. 10 μM CBDV tended to increase phosphorylation at the S800 site of TRPV1.	Iannotti et al. (2014)
Cannabichromene (CBC)	Cannabichromene	1‐μM 24‐h ATP assay/viability and differentiation for 2 days	Neuroprotection	Adult neural stem cells/progenitor cells (NSPC)	*n* = 6	CBC raised viability in B27 medium. CBC had no significant effect on proliferation. In B27 medium, CBC up‐regulated nestin, but reduced GFAP.	Shinjyo & Di Marzo (2013)
Cannabinol (CBN)	Cannabinol/Δ^8^ THC	100, 20, 4, 0.8, 0.16, or 0 μM for 48 h	Huntington's disease	PC12 cells expressing polynucleotide repeats (103 glutamines)	*n* = 2 repeats, average 3– 4 wells	Cannabinol reduced LDH activity in medium at 20 and 100 μM. At 100 μM, CBN decreased LDH release by 84%. Protective EC_50_ of CBN was determined to be 30 μM in this model.	Aiken, Tobin, & Schweitzer, 2004
	Cannabinol (+THC and CBD)	0.1, 1, 2.5, 5, and 10 μM for 24 h	Oxidative stress and neuroprotection	Primary cerebral granule cells (rats/mice), CB1 expressing cell lines. PC12 and HT22 cell lines	*n* = 3	Cannabinol was shown to be a potent antioxidant.	Marsicano, Moosmann, Hermann, Lutz, and Behl (2002)
Tetrahydrocannabidivarin (∆^9^‐THCV)	∆^9^‐THCV	0, 5, 10, 20, 40, and 50 μM applied directly after epileptiform activity. 20‐min pretreatment at 10 μM	In vitro electrophysiology (epileptiform bursting)	Brain slices obtained from male and female outbred rats	*n* = 5	∆^9^‐THCV (20–50 μM) decreased burst incidence, PDS amplitude and frequency. The most significant effect was at 50 μM. ∆^9^‐THCV also decreased epileptiform burst speed (40 μM). ∆^9^‐THCV was found to act as a CB_1_ ligand in receptor binding assays.	Hill et al. (2010)
Tetrahydrocannabinolic acid (∆^9^‐THCA)	∆^9^‐THCA	0.01, 0.1, 1, and 10 μM for 48 h	Parkinson's disease	Dopaminergic neuronal cell culture	*n* = 3–4 wells/treatment	∆^9^‐THCA had no effect on the survival of dopaminergic neurons, but at 10 μM led to an increased cell count (123%) and morphology was ameliorated versus control cultures.	Moldzio et al. (2012)
Mixed	∆^9^‐Tetrahydrocannabinolic acid (∆^9^‐THCA) and cannabidiolic acid (CBDA), cannabigerol (CBG)	0, 0.5, and 1 μM (∆^9^‐THCA) N2a cells—48 h. 0 and 0.1–15 μM (∆^9^‐THCA, CBDA, and CBGA in HEK‐293 T cells)—6 h. 1–10 μM ∆^9^‐THCA STHdh^Q7/Q7^ cells—1 h/CB	Huntington's disease/neurodegeneration	HEK‐293 T Neuro‐2a STHdh^Q7/Q7^ And STHdh ^Q111/Q111^ cells	*n* = 5 repeats	∆^9^‐THCA increased neuronal cell viability post serum deprivation and increased mitochondrial mass. This effect was blocked by a PPARγ antagonist GW9662. All cannabinoid acids induced PPARγ transcriptional activity in HEK293 cells.	Nadal et al. (2017)
	Cannabichromene, cannabidiol, cannabidivarin, cannabigerol, cannabinol, ∆^9^‐tetrahydrocannabinol, ∆^9^‐tetrahydrocannabinolic acid	0, 0.1, 1, and 10 μM for 48 h	Neuroprotection	N18TG2 cells (neuroblastoma cell line)	In triplicate with 2–5 repeats	Emerging phytocannabinoids did not affect the number of dopaminergic neurons. CBG and CBC decreased GSH levels (0.1 and 1 μM and 1 and 10 μM). 0.1 μM CBDV reduced GSH levels by 9.6%; THC, THCA, and CBN have no effect. CBDV and CBN decreased resazurin reduction at 10 μM (32.9 and 38.9%) and affected PI uptake at all concentrations. CBG also affected PI uptake at 0.1 and 10 μM.	Rosenthaler et al. (2014)
	Cannabigerol, cannabichromene, cannabidivarin, and cannabinol (as well as THC, CBD, and CBD derivative DMCBD*)	250 nM–10 μM Oxytosis assay, 30 min. Energy loss assay: 22 h. Trophic factor withdrawal, 48 h	Alzheimer's disease	MC65 cells (human nerve cell line), Ht22 cells (mouse hippocampal cell line), and BV2 microglial cell line	*n* = 6 (twice in triplicate)	CBG, CBDV, CBC, CBN, and THCA prevented oxytosis. CBG, CBDV, CBC, and CBN preserved trophic factors. THCA was toxic to MC65 cells at 1 μM; however, CBDV, CBC, CBN, and CBDA prevented amyloid toxicity at ≤100 nM. CBDV, CBG, CBC, and CBN (100 nM) prevented MC65 neurons from accumulating amyloid β (Aβ).	Schubert et al. (2019)

^a^ Results from 3 independent experiments.

^b^ For in vivo data see Table 2.

**TABLE 2:** Summary of included *in vivo* studies

Phytocannabinoid	Compound	Dose/route/time	Neuro model	Animals^a^	*n* number	Results	Study
Cannabigerol (CBG)	Cannabigerol derivatives VCE‐003 and VCE‐003.2	10 mg·kg^−1^ of body weight intraperitoneally per day until kill	Two models of Huntington's disease	**M** CD1 mice (12 weeks)	*n* = 7 each group	QA model: VCE‐003.2 RotaRod performance, prevented neuronal loss, microglial activation and reduced astrogliosis. 3NP model: VCE‐003.2 improved motor deficits, reduced all pro‐inflammatory mediator release, and prevented neuronal loss.	Díaz‐Alonso et al. (2016)
	Cannabigerol derivative VCE‐003.2	10 mg·kg^−1^ oral once daily for 3 days before kill	Huntington's disease	**M** C57/6 N mice (10 weeks)	*n* = 3–6 mice/condition	VCE‐003.2 promoted neurogenesis, increased GFAP‐positive cells, and reduced microglial activation. Mice performed better on the RotorRod test drug treated versus vehicle.	Aguareles et al. (2019)
	Cannabigerol derivative VCE‐003.2	Oral 10 mg·kg^−1^, 20 mg·kg^−1^, 16 h after LPS for 28 days daily	LPS‐induced Parkinson's disease	C57BL/6 **F** mice, 7–11 months old	*n* = 6 mice per group	20 mg·kg^−1^ partly corrected altered cylinder rearing test but poor activity in rotarod and CAA tests. VCE—003.2 attenuated TNF‐α, IL‐1β (greatest effect at 20 mg·kg^−1^) and recovered TH nigrostriatal neurons.	Burgaz, García, Gómez‐Cañas, Muñoz, and Fernández‐Ruiz (2019)
	Cannabigerol derivative VCE‐003	Daily 5 mg·kg^−1^ i.p. for 21 days	Autoimmune encephalomyelitis (EAE) to model MS	**F** C57BL/6 mice	*n* = 6 animals per group	5 mg·kg^−1^ of VCE‐003 decreased EAE symptoms. VCE‐003 decreased microglial/macrophage activation, reduced demyelination, maintained myelin structure, and reduced axonal damage lesions. Significant decrease in all measured inflammatory mediators.	Carrillo‐Salinas et al. (2014)
	VCE‐003 cannabigerol quinone derivative	15 days 5 mg·kg^−1^ i.p. VCE‐003 treated 60 days after infection	Multiple sclerosis (MS) induced by TMEV	SJL/J mice	*n* = 12	Clinical score (0–5) was significantly improved with VCE‐003 treatment. VCE‐003 completely recovered motor activities to normal levels.	Granja et al. (2012)
	VCE‐003.2 cannabigerol derivative	10 mg·kg^−1^ i.p. 16 h post LPS and then daily for 21 days	Parkinson's disease model—LPS induced	**M** C57BL/6 mice	*n* = 4–6 subjects per group	VC‐003.2 prevented nigrostriatal neuronal loss and reduced microgliosis. Elevation in iNOS was decreased by VC‐003.2 versus control.	García et al. (2018)
	Cannabigerol	50–200 mg·kg^−1^ i.p. 1 h before PTZ seizures	PTZ seizure model (85 mg·kg^−1^ i.p.)	**M** Wistar Kyoto rats	*n* = 72	CBG had no effect on seizure severity, incidence, or timing and did not alter animal mortality. CBG displayed no anti‐convulsant effects.	Hill et al. (2014)
	Cannabigerol derivative VCE‐003	10 mg·kg^−1^ i.p. animals 60 days old up to age 18 weeks	Amyotrophic lateral sclerosis	**M** B6SJL‐Tg (SOD1*G93A) 1Gur/J versus WT	*n* = 5–6 animals per group	In SOD1 mice, VCE‐003.2 delayed disease progression and reduced a number of neuropathological signs. Weight loss was reduced, as were anomalies in clinical score.	Rodríguez‐Cueto et al. (2018)
	Cannabigerol (CBG)	4 intraperitoneal injections every 24 h at a dose of 10 mg·kg^−1^ for 6 weeks (4 weeks after birth to 10 weeks)	Huntington's disease induced by 3NP/R6/2 variant mice	16‐week‐old **M** C57BL/6 mice/4‐ to 10‐week‐old R6/2 mice	*n* = 6–8 animals/experiment	CBG improved motor activities, prevented neuronal loss, increased GFAP staining, and decreased Iba‐1 staining. CBG down‐regulated Huntington associated genes and decreased inflammatory mediators.	Valdeolivas et al. (2015)
Cannabidivarin (CBDV)	Cannabidivarin (CBDV)	Pretreatment vehicle versus 400 mg·kg^−1^ CBDV oral for 3.5 h	Seizures induced by PTZ 95 mg·kg^−1^	Wistar‐Kyoto rats (3/4 weeks old).	*n* = 51	400 mg·kg^−1^ CBDV significantly decreased seizure severity and increased latency to first signs of seizure. CBDV did not significantly affect gene expression changes induced by PTZ.	Amada et al. (2013)
	Cannabidivarin (CBDV)	50, 100, and 200 mg·kg^−1^ i.p. injection 1 h/30 min before induced seizures. 400 mg·kg^−1^ oral gavage 13.5/3.5 h before intraperitoneal PTZ	Epilepsy (mES seizures; 30 mA, 100 Hz for 200 ms, or generalized seizures 85 mg·kg^−1^ PTZ injected intraperitoneally	**F/M** adult Wistar Kyoto rats. Non‐Agouti (DBA/) mice 3–4 weeks, ICR (CD‐1) mice 5 weeks old	*n* = 80 (10/group). 640 Wistar rats 3–4 weeks old (*n* = 15/group)	200 mg·kg^−1^ CBDV—90% of mice remained seizure free. In rats, CBDV significantly decreased PTZ seizure severity and rodent mortality (200 mg·kg^−1^) and delayed seizure onset. On co‐administration experiments, 2.9% of rats (*n* = 7) exhibited a fatal reaction to CBDV administration.	Hill et al. (2012)
	Cannabidivarin (only data from purified CBDV is reported here)	1 h pretreatment 50, 100, and 200 mg·kg^−1^ i.p. (rats) 10–200 mg·kg^−1^ i.p. (mice)	PTZ seizures (85 mg·kg^−1^) or pilocarpine (380 mg·kg^−1^).	**M** Wistar Kyoto rats, **M** MF1 mice, DBA/2 mice 3–4 weeks	*n* = 10 mice *n* = 15 rats	CBDV significantly affected observed seizure severity >50 mg·kg^−1^. Mortality was reduced by CBDV administration and suppressed seizure activity (100 mg·kg^−1^)	Hill et al. (2013)
	Cannabidivarin (CBDV)	2, 20, and 100 mg·kg^−1^ versus vehicle control, daily intraperitoneally for 14 consecutive days	Rett syndrome	5‐month‐old MeCP2–308 (B6.129S‐MeCP2tm1Heto/J	*n* = 70	20 mg·kg^−1^ CBDV improved motor learning ability. Brain weight was increased with CBDV treatment. CBDV had no effect on GPR55 levels and neurotrophin levels.	Vigli et al. (2018)
	Cannabidivarin (96.4% CBDV, 3.6% CBD; started on postnatal day 28, lasting until day 67)	0.2, 2, 20, and 200 mg·kg^−1^ i per day i.p. initiated postnatal day (PND) 28 until PND 67.	Rett syndrome model; (WT vs. Mecp2 KO)	Mecp2–mouse (WT vs. KO).	*n* ≥ 5 per treatment group total *n* = 112	2–200 mg·kg^−1^ per day CBDV reduced tremors, and 0.2 mg·kg^−1^ per day was ineffective. CBDV reduced hind limb clasping but again not at the lowest dose tested. CBDV improved breathing and gait abnormalities, reduced total symptom score, and improved neurological motor deficits.	Zamberletti et al. (2019)
Cannabichromene (CBC)	CBC	0.01 ml·g^−1^ and 25, 50, and 75 mg·kg^−1^ CBC (mice), 1.0 ml·kg^−1^, 10–75 mg·kg^−1^ CBC (rats) i.p. for 1 h prior to electroshock	Electroshock seizure test: 50 mA intensity for 0.2 s	**M** ICR albino mice or male Sprague–Dawley rats	*n* = 90 (mice), 193 mice, 106 rats	CBC/THC had no effect on tonic hindlimb extension. CBC did not alter latency. CBC (lowest dose) shortened the duration of extension. All doses of CBC depressed motor activity (first 10‐min interval).	Davis and Hatoum (1983)
Cannabinol (CBN)	CBN	5 mg·kg^−1^·day^−1^ subcutaneous pouch (25‐g mouse). 28 days up to 12 weeks	Amyotrophic lateral sclerosis (ALS) SOD1 model	**M** Tg (SOD1‐G93A) 2Gur (11) mice. Assigned 6 weeks of age	*n* = 18	Motor abnormalities were delayed by CBN versus vehicle. No significant difference for PaGE test assessment or the age at which animals reached end stage.	Weydt et al. (2005)
Tetrahydrocannabidivarin (∆^9^‐THCV)	∆^9^‐THCV	2 mg·kg^−1^ i.p. for 14 days	Parkinson's disease (by 6‐hydroxytryptamine‐6‐HT) or LPS	**M** Sprague–Dawley rats/CB2 knockout mice	*n* = 5–6 rats per group	THCV improved motor activities, reduced neuronal loss and reduced microglial activation. THCV was able to preserve TH positive neurons (LPS model).	García et al. (2011)
	∆^9^‐THCV	0.025, 0.25, and 2.5 mg·kg^−1^ i.p. + vehicle prior to initiating seizures	Seizures induced by 80 mg·kg^−1^ PTZ	**M** Wistar rats	64 rats in total; *n* = 16 per group	Median seizure severity, duration, progression, or latency was unaffected by any dose of THCV. 33% of animals exhibited a complete absence of seizures at a dose of 0.25 mg·kg^−1^ THCV.	Hill et al. (2010)
Tetrahydrocanvnabidiolc acid (∆^9^‐THCA)	∆^9^‐THCA	20 mg·kg^−1^ i.p. 30 min before 3NPA, every 24 h for 4 days	Huntington's disease (3 NPA model)	**M** C57BL/6 mice	*n* = 70; 9 animals per group	THCA improved hindlimb dystonia and locomotor activity. THCA down‐regulated all pro‐inflammatory mediators.	Nadal et al. (2017)

^a^ Animal sex denoted by **F** (female) and **M** (male).

**FIGURE 2:**
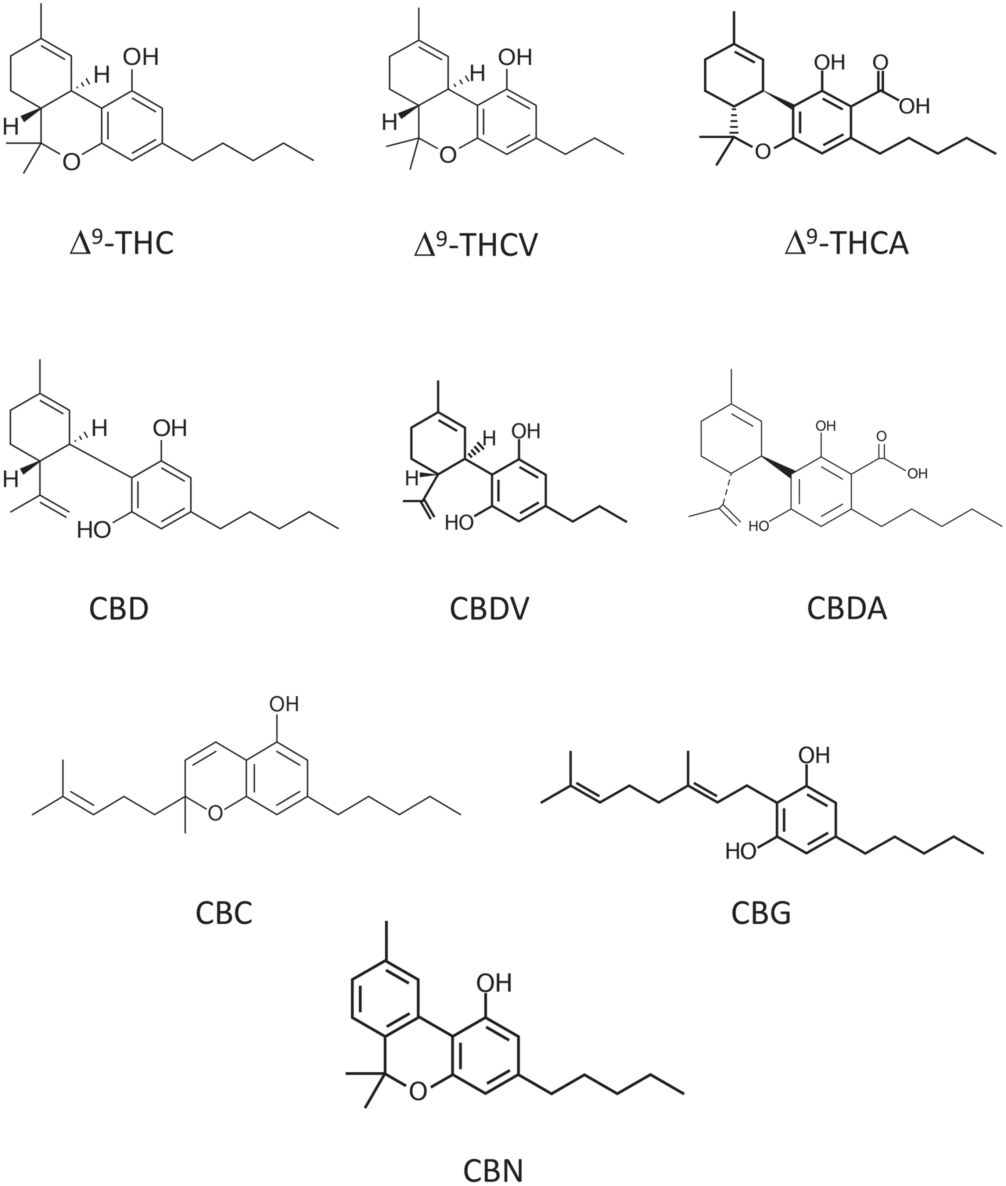
Structured and pharmacological profiles of some of the minor phytocannabinoids with cannabidiol (CBD) and tetrahydrocannabidiol (∆^9^‐THC) included for reference: ∆^9^‐tetrahydrocannabinolic acid (∆^9^‐THCA), ∆^9^‐tetrahydrocannabinolic (∆^9^‐THCV), cannabidivarin (CBDV), cannabidiolic acid (CBDA), cannabichromene (CBC), cannabigerol (CBG), and cannabinol (CBN)

Within the 28 studies, the neuroprotective models were epilepsy (*n* = 7), Huntington's disease (*n* = 6), Parkinson's (*n* = 4), amyotrophic lateral sclerosis (*n* = 3), neuroprotection (not disease specific, *n* = 2), multiple sclerosis (MS; *n* = 1), Rett syndrome (*n* = 2), neuroinflammation (*n* = 1), Alzheimer's (*n* = 1), and oxidative stress (*n* = 1). Fifteen papers studied CBG or its derivatives, five studies used CBN, eight studies used CBDV, and four studies used CBC. Only two studies used Δ^9^‐THCV, and three used Δ^9^‐THCA. CBDA was only included in one study. No data on the neuroprotective effects of CBGA, CBGV, CBCA, CBCV, CBCVA, CBGVA, or CBDVA were identified. Figure 2 shows some of the minor phytocannabinoids structures with CBD and Δ^9^‐THC for reference, and Table 3 summarises the neurological conditions for which emerging cannabinoids have shown therapeutic potential.

**TABLE 3:** Summary of the conditions where emerging cannabinoids have been studied

	Cannabigerol (CBG)/derivatives	Cannabidivarin (CBDV)	Cannabichromene (CBC)	Cannabinol (CBN)	Cannabidiolic acid (CBDA)	∆^9^‐THCV	∆^9^‐THCA
Huntington's	✓	‐	‐	✓	X	‐	✓ PPARγ^a^
Multiple sclerosis	✓	‐	‐	‐	‐	‐	‐
Autoimmune encephalomyelitis	✓PPARγ/CB_2_ ^a^	‐	‐	‐	‐	‐	‐
Parkinson's	✓PPARγ^a^	‐	‐	‐	‐	✓	✓
Neuroinflammation /neuroprotection	✓	✓	✓	✓	‐	✓	✓
Epilepsy/seizure	✗	✓TRPV1^a^	✓	‐	‐	✓	‐
Amyotrophic lateral sclerosis (ALS)	✓	‐	‐	✓	‐	‐	‐
Oxidative stress	‐	‐	‐	✓	‐	‐	‐
Rett syndrome	‐	✓	‐	‐	‐	‐	‐
Alzheimer's disease	✓	✓	✓	‐	‐	‐	‐

*Note.* A tick or cross represents whether a cannabinoid showed efficacy in a condition or not. A dash means that a cannabinoid has yet to be studied in a condition.

^a^ Some of the compounds neuroprotective effects were mediated by this receptor, but no other receptors were probed.

### 3.1 Cannabigerol (CBG) and its derivatives

Nine studies included *in vitro* data, and eight included *in vivo* data on CBG and its derivatives that are formed by the oxidation of CBG (Carrillo‐Salinas et al., 2014; Díaz‐Alonso et al., 2016; García et al., 2018). VCE‐003 and VCE‐003.2 have displayed increased affinity for PPARγ, thus maintaining their anti‐inflammatory properties while having little affinity for CB_1_ and CB_2_ receptors (VCE‐003 *K*_*i*_ > 40 μM for CB_1_ and *K*_*i*_ > 1.76 μM CB_2_, Granja et al., 2012, and VCE‐003.2 *K*_*i*_ > 40 μM for both CB_1_ and CB_2_, García et al., 2018). All studies except one reported a positive effect of CBG, VCE‐003, or VCE‐002.3, compared with control in the disease model being studied. In an *in vivo* model, using 3‐nitropropionic acid (3‐NP) to induce Huntington's disease, CBG (10 mg·kg^−1^ per day i.p.) significantly attenuated the up‐regulation of COX‐2, iNOS, IL‐6, and TNF‐α (Valdeolivas et al., 2015). CBG treatment also prevented 3‐NP‐induced neuronal loss, recovered catalase, SOD and GSH, compared with control, and down‐regulating genes that were directly associated with Huntington's disease including sgkl, Cd44, and normalized levels of huntingtin‐associated protein‐1. Aggregation of mutant Huntingtin protein was diminished, and motor deficits such as hindlimb clasping and dystonia and general locomotor activity were also improved (Valdeolivas et al., 2015). Hill et al. (2014) assessed the anti‐convulsant potential of CBG (50–200 mg·kg^−1^ i.p.) when administered prior to the initiation of pentylenetetrazole (PTZ) seizures; however, despite being able to block Nav channel activity, CBG had no effect on seizure severity. No antagonist experiments were conducted in these studies, but Valdeolivas et al. (2015) did show that CBG dose‐dependently activated PPARγ in cultured striatal cells (WT and mutant huntingtin; supplementary data). Four studies reported that the CBG derivatives VCE‐003 (5 mg·kg^−1^ i.p.) and VCE‐003.2 (10 mg·kg^−1^ p.o./i.p.) successfully reduced immune cell activation in macrophages, microglia, and infiltrating neutrophils in models of EAE (to model MS) and Huntington's and LPS‐induced Parkinson's disease (PD; Aguareles et al., 2019; Carrillo‐Salinas et al., 2014; Díaz‐Alonso et al., 2016; García et al., 2018). In the in vivo Parkinson's disease model, García et al. (2018) found that PPARγ antagonist T0070907 (5 mg·kg^−1^) blocked VCE‐003.2‐mediated decreases in TNF‐α, IL‐1β, and iNOS mRNA levels, but no other antagonists were investigated. In a follow‐up study by the same group, 20 mg·kg^−1^ (but not 10 mg·kg^−1^) oral VCE‐003.2 promoted a trend towards recovery in the basal ganglia of LPS‐lesioned mice and was associated with decreases in IL‐1β gene expression, lysosomal‐associated membrane protein‐1 (LAMP‐1), and glial fibrillary acidic protein (GFAP) immunostaining (Burgaz et al., 2019). Orally dosed VCE‐003.2 (10 mg·kg^−1^) promoted neurogenesis in mice subjected to mutant Huntingtin expression in a Huntington's disease model (Aguareles et al., 2019). In another model of Huntington's disease VCE‐003.2 (10 mg·kg^−1^ i.p.) prevented neuronal loss, indicated by increases in Nissl and NeuN staining and at the same dose improved RotaRod performance and reduced astrogliosis in mice, measured by attenuated levels of GFAP and ionized calcium binding adaptor molecule 1 (Iba‐1; Díaz‐Alonso et al., 2016). Rodríguez‐Cueto et al. (2018) found that VCE‐003.2 10 mg·kg^−1^ i.p. successfully improved neuropathological deterioration and normalized CB_2_ receptor and IL‐1β levels, in an experimental model of amyotrophic lateral sclerosis, but again no mechanisms of action were probed.

*In vitro*, Schubert et al. (2019) reported that CBG (100 nM) prevented MC65 neurons from accumulating toxic amyloid β (Aβ) protein in an Alzhiemer's disease model. CBG also preserved neuronal trophic factors in primary rat cortical neurons (EC_50_ 1.5 μM) and prevented oxytosis in mouse HT22 hippocampal nerve cells (EC_50_ 1.9 μM). Although no mechanisms were explored in this study, neither MC65 neurons nor HT22 cells express CB_1_ or CB_2_ receptors, leading the authors to conclude that these effects were mediated independently of these receptors. In N2a cells, VCE‐003.2 (10 and 25 μM) prevented excitotoxicity induced by glutamate and in models of LPS induced Parkinson's disease and amyotrophic lateral sclerosis (García et al., 2018; Rodríguez‐Cueto et al., 2018). Similarly, VCE‐003 (0.1–25 μM) dose dependently protected neuronal cells in a model of MS, while VCE‐003.2 (500 nM) promoted neuronal differentiation when dosed for 21 days in an *in vitro* model of Huntington's disease, but no antagonist experiments were conducted to explain these effects (Aguareles et al., 2019; Granja et al., 2012). In a model of neuroinflammation, pretreatment with CBG (7.5 μM) improved viability in cells treated medium from LPS‐stimulated macrophages and, while authors reported that CBG treatment resulted in PPARγ down‐regulation, no direct mechanistic probing was conducted (Gugliandolo, Pollastro, Grassi, Bramanti, & Mazzon, 2018). Granja et al. (2012) and Carrillo‐Salinas et al. (2014) found that treatment with VCE‐003 (1 and 2.5 μM and 1, 10, and 25 μM) blocked the secretion of a number pro‐inflammatory mediators including IL‐6, TNF‐α, IL‐1β, and CCL3 in macrophages and primary microglia. VCE‐003.2 also attenuated TNF‐α and L‐1β secretion but from BV2 mouse microglial cells (5 μM) and astroglial cells (1 and 5 μM; Díaz‐Alonso et al., 2016; García et al., 2018; Rodríguez‐Cueto et al., 2018). Díaz‐Alonso et al. (2016) and García et al. (2018) deduced that VCE‐003.2 did not mediate its protective effects via CB_1_ or CB_2_ receptors due to poor binding affinity (*K*_*i*_ > 40 μM) and both groups found that VCE‐003.2 was an agonist at PPARγ (IC_50_ of 1.2 μM).

### 3.2 Cannabidivarin (CBDV)

All *in vivo* cannabidivarin (CBDV) studies evaluated the anti‐epileptic properties of the compound in models of Rett syndrome and MES seizures (Amada, Yamasaki, Williams, & Whalley, 2013; Hill et al., 2012; Hill et al., 2013; Vigli et al., 2018; Zamberletti et al., 2019). Doses in these studies ranged from 0.2 to 400 mg·kg^−1^ per day in rodents with efficacy in reducing tremors was observed between 2 and 200 mg·kg^−1^ per day. Two studies reported that 200 mg·kg^−1^ i.p. CBDV significantly decreased PTZ seizure severity and mortality in rats (A. J. Hill et al., 2012; Hill et al., 2013). Hill et al. (2012) found that 90% of animals remained seizure free at a dose of 200 mg·kg^−1^ CBDV i.p. per day; however, lower concentrations of CBDV were ineffective (0.2 mg·kg), and CBDV had no effect on the severity of pilocarpine convulsions at any tested concentration (50–200 mg·kg^−1^ per day). CBDV (400 mg·kg^−1^ oral gavage) suppressed PTZ seizures, significantly decreasing seizure severity but had no effect on expression of epilepsy related genes (Amada et al., 2013). Another study reported that 20 mg·kg^−1^ i.p. CBDV dosed for 14 days improved brain weight in Rett syndrome (RTT) mice, compared with WT mice, but had no effect on neurotrophin levels (Vigli et al., 2018). None of these *in vivo* studies conducted antagonist experiments to further elucidate the anticonvulsant effects of CBDV.

In HEK293 cells transfected with TRPV1, 2, and 3 channels, CBDV caused a concentration‐dependent bidirectional current at TRPV1 channels similar to capsaicin, and capsazepine (TRPV1 channel antagonist) blocked this effect. Furthermore, 5'‐iodoresiniferatoxin (5'‐IRTX), a selective antagonist of TRPV1 channels counteracted the effect of CBDV in the duration but not amplitude of neuronal burst. These data suggest that CBDV acts as an agonist at these channels, but some of CBDVs effects are mediated independently of this channel. However, no other antagonists were tested to establish which receptors were responsible for the other effects of CBDV (Iannotti et al., 2014). Hill et al. (2012) reported that CBDV (10 and 100 μM) decreased the amplitude and duration of local field potentials in hippocampal brain slices, with an anti‐epileptiform effect observed in the CA1 region (100 μM). CBDV also showed efficacy in an *in vitro* model of Alzheimer's disease, preventing oxytosis and energy loss in HT22 cells (EC_50_ 1.1 μM and 90 nM, respectively), as well as reducing Aβ toxicity (EC_50_ 100 nM) and trophic withdrawal (EC_50_ 350 nM); however, no mechanistic data were reported to determine how these effects were mediated (Schubert et al., 2019).

### 3.3 Cannabichromene (CBC)

In a model of electroshock seizure, CBC (10–75 mg·kg^−1^ i.p. per day) significantly depressed motor activity during the first 10‐min interval, but subsequently only the highest dose was effective (Davis & Hatoum, 1983). *In vitro*, Shinjyo and Di Marzo (2013) found that 1μM CBC increased viability of adult nestin‐positive neuronal stem cells when applied in medium without growth factors (B27 medium), by inducing ERK phosphorylation. No antagonist data were presented in these studies.

### 3.4 Cannabinol (CBN)

Only one *in vivo* study assessed CBN (5 mg·kg^−1^ per day) in an SOD1 model of amyotrophic lateral sclerosis. CBN delayed motor abnormalities at Day 17 in the chronic treatment regimen, compared with vehicle control, but disease progression was not affected (Weydt et al., 2005). In a model of Huntington's disease, Aiken et al. (2004) found that CBN reduced LDH activity in PC12 cells (20 and 100 μM), but the authors did not investigate the mechanism(s) of this effect. CBN displayed potent antioxidant activity in primary cerebral granule cells under oxidative stress conditions; however, no antagonist data were presented on this cannabinoid (Marsicano et al., 2002).

### 3.5 Δ^9^‐THCV

Male Sprague–Dawley rats and CB_2_ receptor knockout mice were dosed with 2 mg·kg^−1^ per day Δ^9^‐THCV over a period of 14 days in a model of Parkinson's disease, induced by 6‐hydroxydopamine (6‐OHDA) or LPS (García et al., 2011). Δ^9^‐THCV reduced slow motor movements induced by 6‐OHDA and enhanced mean velocity of movement with a potency similar to rimonabant. Chronic Δ^9^‐THCV dosing reduced microglial activation and preserved nigrostriatal dopaminergic neurons after 6‐OHDA application and in the LPS model of Parkinson's disease, Δ^9^‐THCV preserved TH positive neurons, mirroring the effects of the CB_2_ receptor agonist HU‐308. Thus, authors speculated that Δ^9^‐THCV mediated at least some of its effects in the LPS model via CB_2_ receptors (García et al., 2011). Also, 2 mg·kg^−1^ Δ^9^‐THCV blocked the effects of the CB_1_ receptor agonist, CP55,940, suggesting it acts as an antagonist at this receptor. However, no data were presented assessing if such antagonistic properties of Δ^9^‐THCV at CB_1_ receptors mediated its protective effects in the 6‐OHDA or LPS models of Parkinson's disease. Hill et al. (2010) studied Δ^9^‐THCV in a seizure model induced by 80 mg·kg^−1^ PTZ and found that at a dose of 0.25 mg·kg^−1^ i.p. Δ^9^‐THCV, with 33% of animals having a complete absence of seizures. Although no direct mechanistic probing was investigated, receptor binding assays were performed on rat cortical membranes, and Δ^9^‐THCV was found to act as a CB_1_ receptor ligand (CB_1_ *K*_i_ ∼ 290 nM; [^3^H]SR141716A displacement but no agonist stimulation using [^35^S] GTPγS binding; Hill et al., 2010).

### 3.6 Δ^9^‐THCA

In an acute 3‐NP model of Huntington's disease, Nadal et al. (2017) observed a significant improvement in hindlimb dystonia (uncontrollable hindlimb muscle contraction) and locomotor activity in male, C57BL/6 mice treated with Δ^9^‐THCA (20 mg·kg^−1^ per day i.p.). Δ^9^‐THCA also prevented astrogliosis and microgliosis and attenuated the up‐regulation of pro‐inflammatory mediators induced by 3‐NP. These effects were blocked when mice were co‐administered with the PPARγ antagonist T0070903 (with the exception of IL‐6; Nadal et al., 2017 *).* *In vitro*, N2a cells infected with the huntingtin polyQ repeats resulted in toxicity, which was significantly reduced by treatment with ∆^9^‐THCA, as well as decreased expression of inflammatory mediators: TNF‐α, iNOS, IL‐6, and COX‐2. Δ^9^‐THCA also improved neuronal viability post‐serum deprivation, and this effect was prevented by GW9662, a PPARγ antagonist. No other antagonists were used in this study (Nadal et al., 2017 *).*

Δ^9^‐THCA (0.01–10 μM) displayed no pro‐survival effect on dopaminergic neurons but had a significant, positive effect on cell count (123%) when compared to the control, in an *in vitro* model of Parkinson's disease (Moldzio et al., 2012).

## 4 DISCUSSION

To our knowledge, this is the first systematic review on the neuroprotective effects of lesser‐known, minor phytocannabinoids in various models of neurological disease. Data obtained from our search revealed that CBG, VCE.003, VCE.003.2, and CBDV were the most promising candidates as neuroprotectants, while Δ^9^‐THCV, Δ^9^‐THCA, CBC, and CBN have limited but encouraging data as neuroprotectants. CBG, VCE.003, VCE.003.2, and Δ^9^‐THCA mediated their neuroprotective effects at least in part by the nuclear receptor PPARγ. CBDV was found to mediate some of its antiepileptic effects via TRPV1 channels, and Δ^9^‐THCV was found to be a CB_1_ receptor ligand and a possible CB_2_ receptor agonist, but no experiments were conducted to establish whether its neuroprotective action was mediated by CB_1_ or CB_2_ receptors. No other receptors were investigated, and no studies assessed the neuroprotective potential of CBDA, CBGA, CBGV, CBCV, CBGVA, or CBDVA.

CBG was first isolated in 1964 by the same group that reported the structure of Δ^9^‐THC (Gaoni & Mechoulam, 1964). It exhibited antioxidant and anti‐inflammatory properties, while displaying no psychotropic effects, as it is a poor CB_1_ receptor agonist (Gauson et al., 2007; Navarro et al., 2018; Rosenthaler et al., 2014). CBG is a partial agonist at CB_2_ receptors, a potent α_2_‐adrenoceptor agonist (EC_50_ 0.2 nM) and a moderate 5‐HT_1A_ receptor antagonist, as well as interacting with various TRP isoforms including TRPV1 and 2 channels (Cascio, Gauson, Stevenson, Ross, & Pertwee, 2010; De Petrocellis et al., 2012). Studies included here show that these compounds have significant anti‐inflammatory effects, including attenuating cytokine release and decreasing the activation of immune cells, an effect observed in both *in vitro* and *in vivo* models.

CBG and its derivatives were particularly effective in models of Huntington's disease, targeting multiple facets of the disease including gene expression, easing motor symptoms, reducing microglial activation, and attenuating the inflammatory response. Huntington's disease pathophysiology, like other neurodegenerative disorders, exhibits uncontrolled microglial activation, which is a key part of the neuroinflammatory response. In early stages of this disease, PET imaging has revealed marked microglial activation, which was correlated with impairments of neuronal activity (Tai et al., 2007). Microglial activation along with increases in pro‐inflammatory mediators has also been detected in post‐mortem Huntington's disease brains (Palpagama, Waldvogel, Faull, & Kwakowsky, 2019). Interestingly, microglial mediated neuroinflammation was suppressed with the activation of CB_2_ receptors (Ehrhart et al., 2005). However, given VCE‐003 and VCE.003.2's protective effects were likely to be CB_1_ and CB_2_ receptor‐independent, their effects on microglial activation are likely to be via a different mechanism, possibly through the activation of PPARγ, which has an important role in regulating the inflammatory response, especially in the CNS (see Bright, Kanakasabai, Chearwae, & Chakraborty, 2008; Villapol, 2018). It is also worth noting that microglial activation can be protective, preserving neurons by secreting anti‐inflammatory cytokines such as IL‐4 and IL‐10 as well as various trophic factors (see Le, Wu, & Tang, 2016, and Pöyhönen, Er, Domanskyi, & Airavaara, 2019). In line with these observations, there effectively needs to be a balancing act between enabling some degree of microglial activation to protect neurons, while limiting their overactivation that would ultimately lead to damage. Given that the symptoms of Huntington's disease are currently managed using VMAT inhibitors (such as TBZ) to decrease levels of monoamines, it would be useful to assess whether CBG and its derivates have any efficacy as VMAT inhibitors, or whether their protective effects in models of Huntington's disease are independent of this mechanism. If the latter is the case, future studies should investigate low‐dose VMATs (to minimize neuropsychiatric side effects) together with CBG or its derivatives as an adjuvant therapy to assess if there is an additive, or even synergistic, protective effect of these compounds.

Long‐term dose tolerability and a lack of accumulation in tissue are both essential features of neuroprotective agents as these drugs are typically taken for life after disease onset. In a study conducted by Deiana et al. (2012), CBG was found to have similar PK profiles in rats and mice but exhibited slower brain penetration in mice. Both animals also had higher concentrations of CBG following i.p. injection compared to oral administration, but interestingly in rats, this did not equate to higher concentrations in brain tissue (Deiana et al., 2012). From the results in our review, treatment with CBG, VCE‐003, and VCE.003.2 was well tolerated and ranged from just 3 days to 10 weeks with two studies dosing CBG orally and seven studies dosing intraperitoneally. Deiana et al. (2012) reported that animals tolerated CBG better after i.p. administration, compared with the oral route. In humans, i.p. dosing is not a viable means of regular administration, and all drugs given orally have a larger side effect profile. Moreover, patients receiving certain oral therapies for neurological conditions, such as levodopa for Parkinson's disease, must also take medications to minimize peripheral effects (Fahn, 2008). Therefore, dose formulation and route of administration for these compounds should be carefully assessed, based on thorough ADME profiling and feasibility of long‐term dosing.

CBG exhibited positive effects in two Huntington's disease models, despite one study using oral and the other i.p., administration. Of note, CBD has already been trialled in Huntington's disease patients; CBD (10 mg·kg^−1^; 700 mg average daily dose) was given for 6 weeks and resulted in a consistent plasma level of 5.9–11 ng·mL^‐1^. Once treatment had stopped, elimination was between 2 and 5 days, suggesting CBD did not accumulate and remain in plasma longer than 5 days in these Huntington's disease patients (Consroe et al., 1991). Further studies should elucidate whether CBG and its derivatives display efficacy in humans and clarify whether their activation of PPARγ corresponds to their neuroprotective properties and if other receptors are involved. More data are also needed on the PK profiles of CBG and its derivatives in older mice and larger mammals and to establish whether it exhibits a similar elimination to CBD in humans. These factors would aid in the translation of this compound as a treatment for neurodegenerative conditions.

Cannabidivarin (CBDV) is a structural analogue of CBD, with the molecule shortened by two methylene bridges (Morales, Hurst, & Reggio, 2017; Vollner, Bieniek, & Korte, 1969). From our search, *in vivo* studies consistently reported 200 mg·kg^−1^ i.p. CBDV having anti‐epileptic effects and a 400 mg·kg^−1^ oral dose also showing promise. Like CBD, CBDV is a agonist at TRPV1/2 and TRPA1 channels, and an antagonist at TRPM8 channels, which may explain similarities in their neuroprotective properties, particularly the action of CBDV as an agonist at TRPV1 channels (De Petrocellis et al., 2011; Iannotti et al., 2014; Scutt & Williamson, 2007). In our review, studies showed that CBDV did not affect neurotrophic levels or epilepsy‐related gene expression. Thus, it can be assumed that CBDV mediates its protective effects independent of these pathways (Amada et al., 2013; Vigli et al., 2018). Deiana et al. (2012) reported that CBDV was rapidly absorbed in mice and rats, but there was a higher drug concentration in plasma and brain following oral treatment in rats compared to mice. Furthermore, while i.p. injection resulted in similar PK profiles in the two species, brain concentrations in rats were higher. This brings into question the differences in the amount of CBDV delivered to the brain in the studies conducted in mice compared with rats presented in this review and whether this influenced study outcomes. Only two studies reported chronic CBDV dosing both in models of Rett syndrome, highlighting the need for future studies to assess the long‐term tolerability of CBDV as an anti‐epileptic agent and how different species exhibit different bioavailability of this compound, as these will both affect the translatability of CBDV to humans.

Although out of the scope of this review, it is worth noting that CBDV has already been trialled as an anti‐convulsant by GW Pharmaceuticals in a phase IIa, placebo‐controlled study of 162 adult patients (clinical trial number: NCT02369471/NCT02365610). The drug GWP42006 (which contains CBDV as its main ingredient) was dose titrated (over 2 weeks) up to a 800 mg twice daily dose for a 6‐week stable treatment period. However, focal seizures were inadequately controlled with this dose and GWP42006 displayed no difference in efficacy to the placebo control group (Schultz, 2018). While this may cast doubt on the translatability of the evidence presented in this review, it is worth highlighting that the maximum dose in humans from the GW study would be considerably less than if the same dose regimens as the *in vivo* studies were followed for a 60‐kg human. Furthermore, Morano et al. (2020) have suggested that the inability of CBDV to control seizures was in part due to an extremely high response from the placebo group and that the use of purified CBDV may have also influenced the study outcome. Therefore, it is important to exercise caution when extrapolating the findings from the *in vitro* and *in* *vivo* data presented here and what doses may be effective in clinical trials.

Cannabichromene (CBC) was first isolated in 1966 by Gaoni and Mechoulam and is a non‐psychotropic cannabinoid that does not interact with CB_1_ receptors (Gaoni & Mechoulam, 1966). CBC is an agonist at CB_2_ receptors and TRP channels, acting potently at TRPA1 as well as displaying some activity at TRPV3 and TRPV4 channels (Cascio & Pertwee, 2015; De Petrocellis et al., 2008, 2011; de Petrocellis et al., 2012; Udoh, Santiago, Devenish, McGregor, & Connor, 2019). CBC (0.001–1 μM) exhibited promising anti‐inflammatory effects in an *in vitro* model of colitis, decreasing LPS increased nitrite levels and attenuating IFN‐γ and IL‐10 secretion in peritoneal macrophages (Romano et al., 2013). More recently CBC acted as a CB_2_ receptor agonist in AtT20 cells transfected with these receptors and was confirmed by application of the CB_2_ receptor antagonist AM630, which blocked the effects of CBC (Udoh et al., 2019). We found only two papers related to neuroprotective effects of CBC; *in vivo* CBC suppressed motor activity while *in vitro* CBC improved viability of neural stem cells (Davis & Hatoum, 1983; Shinjyo & Di Marzo, 2013). The anti‐inflammatory effects of CBC may play a pivotal role in its ability to act as a neuroprotectant, as inflammation and overactivation of the immune response are important features of neurodegenerative conditions.Thus, further research should assess this compound in neuro‐inflammatory conditions, where it may have potential.

Cannabinol (CBN) is an oxidation product of ∆^9^‐THC and was the first cannabinoid to be discovered and isolated (Wood, Spivey, & Easterfield, 1899). Like ∆^9^‐THC, it has been shown to activate CB_1_ receptors (*K*_*i*_ 211.2 nM) but with lower potency, as well as acting as an agonist at TRPV2 channels (Rhee et al., 1997; Russo & Marcu, 2017). CBN (1 mg·mL^−1^) was recently shown to reduce mechanical sensitization and sensitivity of afferent muscle fibres in an *in vivo* model of myofascial pain, but no mechanism of action was investigated (Wong & Cairns, 2019). From our search, limited data showed that CBN decreased cell damage and acted as a potent antioxidant in a cell‐based Huntington's disease model (Aiken et al., 2004). The antioxidant activity of CBN is a characteristic feature of cannabinoids, which as previously mentioned, is thought to be due to the presence of the phenolic ring and carboxyl moieties, as well as the ability to increase antioxidant defences. CBD has already shown extensive antioxidant properties, including increasing the levels and activity of antioxidants, capturing ROS, and transforming them into less active forms, as well as activating nuclear erythroid 2‐related factor (NrF2) that governs the transcription of many antioxidant genes (see Atalay, Jarocka‐karpowicz, & Skrzydlewskas, 2020). Oxidative stress is a key feature of neurodegenerative disorders including Parkinson's and Alzheimer's disease. In the latter condition, Aβ deposits contain a significant number of binding sites for biometals (zinc, copper, and iron) that contribute to oxidative stress in patients (Huang, Zhang, & Chen, 2016; Kozlowski et al., 2009). Furthermore, Alzheimer's disease patients have decreased levels of antioxidant enzymes and increased products of oxidative stress, such as peroxidised lipids and oxidized proteins in brain tissue (Kim et al., 2006; Sultana et al., 2011). Also, large amounts of ROS are generated by reactive microglial cells, with studies showing superoxide produced by microglia directly contributing to the death of dopaminergic neurons in Parkinson's disease (Hernandes, Café‐Mendes, & Britto, 2013). It is clear that more information is needed on the pharmacology of CBN, especially its antioxidant potential. Moreover, the ability of CBDV, CBG, CBC, and CBN to reduce Aβ deposits *in vitro* is also noteworthy and it is clearly of interest to examine the antioxidant and anti‐inflammatory potential of these compounds in Alzheimer's disease models *in vivo* and whether these compounds act through mechanisms, similar to those of CBD.

∆^9^‐THCV is a homologue of ∆^9^‐THC differing by just a propyl side chain, and studies have suggested that ∆^9^‐THCV acts as a CB_1_ receptor agonist, sharing properties with ∆^9^‐THC, albeit with less potency (Gill, Paton, & Pertwee, 1970; Pertwee, 2008). They exhibit similarities in their *in vivo* effects such as inducing catalepsy in mice and ∆^9^‐THC‐like effects in humans (Gill et al., 1970; Hollister, 1974). We found two studies where ∆^9^‐THCV showed promise as an anti‐epileptic agent and protected neurons in two models of Parkinson's disease, while García et al. (2011) suggested ∆^9^‐THCV mediated some of its protective effects by acting at CB_1_ and CB_2_ receptors, the possible mechanisms of action of ∆^9^‐THCV was largely unexplored (García et al., 2011; Hill et al., 2010). In an earlier study, ∆^9^‐THCV displaced [^3^H]CP55940 from specific sites in mouse brain and CHO‐hCB_2_ cell membranes (*K*_i_ values 75.4 nM and 62.8 nM, respectively), and along with data from GTPγS‐binding experiments, the authors concluded ∆^9^‐THCV acted as a CB_1_ and CB_2_ receptor antagonist (Thomas et al. 2005). Other groups have shown ∆^9^‐THCV can block CB_1_ receptor activity in murine cerebellar slices and, at 5.8 μM, increased GABA release from neurons, sharing the same properties as AM251, a CB_1_ receptor antagonist (Ma, Weston, Whalley, & Stephens, 2008; Pertwee, 2008). Thus, while there is evidence to suggest ∆^9^‐THCV mediates some of its protective effects via CB_1_ and CB_2_ receptors, the data remain largely unclear, and there is also a lack of investigation into the potential of ∆^9^‐THCV to act at other known cannabinoid targets.

Microglial activation and the presence of neuroinflammatory factors are well known characteristics of Parkinson's disease and well documented among patients (Mogi et al., 1994; Qian et al., 2011). Moreover, studies have demonstrated that microglial overactivation leads to deleterious effects and the exacerbation of the immune response, especially the release of pro‐inflammatory mediators. As observed with the CBG derivative VC‐003.2, microglial activation was decreased by ∆^9^‐THCV, inducing a protective effect by dampening the immune response. Studies have already demonstrated the ability of CBD to modulate the immune response by acting an agonist of PPARγ and altering NF‐κB signalling, which is up‐regulated in both microglia and astrocytes of Parkinson's disease patients. Furthermore, activation of PPARγ leads to inhibition of NF‐κB signalling and decreases mRNA levels of proinflammatory mediators TNF‐a, IL‐1β, IL‐6, and iNOS (Vallée, Lecarpentier, Guillevin, & Vallée, 2017). Therefore, it would be of interest to determine whether ∆^9^‐THCV is able to reduce microglial activation through the same mechanism as CBD, involving the activation of PPARγ.

Limited pharmacokinetic data on Δ^9^‐THCV have shown it exhibits rapid absorption in rats and mice when administered either i.p. or orally but is rapidly eliminated when orally administered (<1.5 h) compared to i.p administration where its elimination rate is >5 h (Deiana et al., 2012). Interestingly, Δ^9^‐THCV exhibited extensive brain penetration (exceeding plasma levels), regardless of the route of administration, meaning it can effectively cross the BBB. At 24 h, Δ^9^‐THCV was no longer detected, suggesting that it exhibits a lack of accumulation in brain tissue (Deiana et al., 2012). Altogether, these features, along with evidence collected in this study, support Δ^9^‐THCV as a neuroprotective agent. However, clearly, more data with Δ^9^‐THCV are needed, especially to assess safety after chronic dosing and whether this compound exhibits tolerance with long‐term use.

∆^9^‐THCA is the acidic precursor of ∆^9^‐THC, and competition binding assays revealed that this compound was unable to achieve displacement of [^3^H]‐CP55,940 (CB_1_ and CB_2_ receptor agonist) up to 10 μM, suggesting ∆^9^‐THCA exhibits poor affinity for CB_1_ or CB_2_ receptors (McPartland et al., 2017). Results from this study also showed that ∆^9^‐THCA has little efficacy at these receptors as it exhibited no inhibition of forskolin‐mediated cAMP, compared to ∆^9^‐THC that acted as an agonist in this assay. Our search revealed that ∆^9^‐THCA had anti‐inflammatory effects that improved neural viability in a model of Huntington's disease, but interestingly, it did not affect the survival of dopaminergic neurons in a model of Parkinson's disease (Moldzio et al., 2012; Nadal et al., 2017). In a recent study, Anderson, Low, Banister, McGregor, and Arnold (2019) reported that ∆^9^‐THCA had extremely poor brain penetration (an optimistic brain–plasma ratio of 0.15) in both vehicles tested. Furthermore, studies have shown that ∆^9^‐THCA has poor stability and rapidly decarboxylates to ∆^9^‐THC, bringing into question whether the ability of ∆^9^‐THCA to act as a neuroprotectant in the studies presented here is actually due to nearly unavoidable contamination with ∆^9^‐THC (Anderson et al., 2019; McPartland et al., 2017). Overall, these data warrant further investigation into ∆^9^‐THCA as a potential neuroprotective and anti‐inflammatory agent, but with caution, and such studies should include purity data on ∆^9^‐THCA to enhance the robustness of the experimental data.

There were no studies identified in this review that looked at the potential neuroprotective effects of other cannabinoid varins or their acidic forms such as CBGV, CBGVA, CBDVA, CBCV, and CBCVA. This may be due to the lack of commercial availability of these compounds due to their low concentrations in the plant, costly synthetic production or that these compounds are not very stable. CBDA was only used in one study on Huntington's disease, where it had no protective effects. This compound, however, has shown promise in other conditions including breast cancer migration, inflammatory pain and nausea (Bolognini et al., 2013; Rock, Limebeer, & Parker, 2018; Takeda et al., 2012), with groups suggesting that CBDA is 1,000 times more potent at the 5‐HT_1A_ receptor than CBD (Bolognini et al., 2013). Activation of the 5‐HT_1A_ receptor is protective both *in vitro* in Parkinsonian models and *in vivo* in models of hypoxia ischaemia (Miyazaki et al., 2013; Pazos et al., 2013). Although Anderson et al. (2019) concluded that CBDA displayed poor brain penetration in an oil‐based formulation, uptake was increased when CBDA was formulated in a Tween‐based vehicle. Also, CBDA was anti‐convulsant at 10 and 30 mg·kg^−1^ displaying greater potency compared to CBD (100 mg·kg^−1^). These data support CBDA's efficacy in the brain, as well as highlighting its potential as an anticonvulsant (Anderson et al., 2019). Considering these points, CBDA may be also protective in conditions such as ischaemic stroke and Parkinson's disease and warrants further investigation. Recent studies have also shown that CBDA, CBGV, and CBGA interact with various TRP channel isoforms including TRPV1, TRPV2, TRPA1, and TRPM8 channels. Of note, CBGV and CBGA were also potent desensitizers of TRPV3 and TRPV4 channels, respectively (De Petrocellis et al., 2012). While the extent of the role of TRP channels in neuroprotection has yet to be fully understood, these receptors are involved in a wide range of neurological disorders. For example, TRPA1‐deficient mice were more likely to sustain damage post ischaemia and TRPA1 channel activation in Alzheimer's disease may have a crucial role in regulating astrocyte‐mediated inflammation (Lee et al., 2016; Pires & Earley, 2018). Conversely, TRPV1 channel activity has been implicated in epilepsy having a role in neuronal excitability and synaptic transmission (Nazıroglu, 2015). Therefore, CBDA, CBGV, and CBGA interactions at TRP channels may be beneficial in conditions that involve these channels in their pathophysiology.

Translatability of these data and the viability of minor phytocannabinoids as neuroprotectants will also rely on understanding and perhaps manipulating their bioavailability and pharmacokinetic properties. In a recent systematic review conducted by our group, Millar, Stone, Yates, and O'Sullivan (2018) highlighted discrepancies regarding CBD bioavailability, *C*_max_, *T*_max_, and half‐life (*t*_1/2_) in humans depending on the route of administration and formulation and whether CBD was dosed in a fed or fasted state. That being said, studies conducted in piglets (Garberg et al., 2017) and rodents (Hammell et al., 2016; Long et al., 2012) have shown a dose‐dependent relationship between CBD administration and brain and plasma concentrations. Limited data extracted by Millar et al. (2018) showed that administration of CBD in humans also led to dose‐dependent increases in plasma concentrations, suggesting the same may apply to brain concentrations in man.

Information on the human metabolites of CBD, ∆^9^‐THC, and other phytocannabinoids is scarce, with the majority of research focusing on the extensive first pass metabolism of CBD and the identification of its urinary metabolites. Of interest, a patent filed by Mechoulam et al. (2010) described that two major metabolites of CBD, 7‐hydroxy (7‐OH) CBD and 7‐carboxy (7‐COOH), are both anti‐inflammatory and dose dependently inhibit TNF‐а, NO, and ROS. However, these data have yet to be confirmed in academic studies or found to be true of other phytocannabinoids. In addition, the cytochrome P450 (CYP) superfamily is responsible for metabolizing 60%–80% of CNS‐acting drugs, 23% by CYP3A4 and 38% CYP2C19, both of which accept CBD as a substrate (Cacabelos, 2010; Iffland & Grotenhermen, 2017). Altogether, these findings highlight that there are major gaps in the ADME of phytocannabinoids, as well as a lack of identification of metabolites and whether they have biological effects. In phase II trials, the minor phytocannabinoids presented in this review will, in all likelihood, be used alongside current therapies to see if they can augment survival of neurons and/or symptom burden, rather than being used as a single agent. In light of the above, it will be essential to consider the interactions that these compounds may have when administered in conjunction with conventional drug therapies (where they exist) and to establish potential synergistic or deleterious effects. Looking forward, initial ADME data will be essential to determine whether these compounds have true clinical potential and for their subsequent formulation and administration.

## 5 CONCLUSIONS

This review aimed to collate and summarise all current data on the neuroprotective potential of phytocannabinoids other than ∆^9^‐THC and CBD. Despite the lack of studies available in this area, we found that all phytocannabinoids tested displayed neuroprotective properties in a range of disorders. CBG and its derivatives displayed significant anti‐inflammatory effects and were particularly effective in Huntington's disease models. CBDV, ∆^9^‐THCV, and CBC were effective as anti‐seizure agents, while CBN displayed antioxidant activity and ∆^9^‐THCA had anti‐inflammatory effects. CBG and ∆^9^‐THCA, like CBD, mediate their anti‐inflammatory effects through PPARγ. Many of the studies were screening studies that conducted no mechanistic probing, suggesting that research into these compounds is still in its early stages. Extensive pharmacokinetic and pharmacodynamic data in larger mammals are also necessary on these compounds, given that all *in vivo* studies in this review were conducted in mice and rats. This would provide more evidence for the facilitation of these compounds as therapies in humans. Further studies are required to investigate the full neuroprotective potential of these compounds particularly the mechanisms underlying their protective effects, as well as exploring whether their combinations may enhance their capabilities as neuroprotectants. While we have focused on a select number of minor phytocannabinoids, based predominantly on their shared physical and biological similarities to CBD, there are over 100 phytocannabinoids and terpenes present in the *Cannabis* plant that could potentially display neuroprotective potential.

### 5.1 Nomenclature of targets and ligands

Key protein targets and ligands in this article are hyperlinked to corresponding entries in http://www.guidetopharmacology.org, the common portal for data from the IUPHAR/BPS Guide to PHARMACOLOGY (http://www.guidetopharmacology.org), and are permanently archived in the Concise Guide to PHARMACOLOGY 2019/20 (Alexander, Christopoulos et al., 2019; Alexander, Cidlowski et al., 2019; Alexander, Fabbro et al., 2019; Alexander, Mathie, et al., 2019).

## AUTHOR CONTRIBUTIONS

N.S. and S.O.S. wrote the paper. N.S. and A.M. conducted the searches. All authors contributed to editing the paper and approving the final version.

## CONFLICT OF INTEREST

S.O.S. is the Chief owner of CanPharmaConsulting. She is on the advisory board for Artelo Biosciences, as well as acting as the science lead for the Centre for Medicinal Cannabis (CMC). Other authors declare that they have no conflict of interest in relation to this review.
